# Right Sinus of Valsalva Aneurysm Mimicking Right Coronary Artery Ischemia

**DOI:** 10.1055/s-0040-1714056

**Published:** 2020-12-23

**Authors:** Antonio Bivona, Vincenzo Caruso, Samir Shah

**Affiliations:** 1 Department of Cardiac Surgery, Guy and St. Thomas' Hospital, London, United Kingdom; 2 Department of Cardiothoracic Surgery, The Essex Cardiothoracic Centre, Basildon, United Kingdom

**Keywords:** aortic surgery, right coronary sinus, aortic aneurysm repair

## Abstract

An aneurysm of a single sinus of Valsalva is rare. It is usually asymptomatic and rarely discovered, unless it compresses the adjacent cardiac structures, or it presents in association with other pathology. We herein describe a case of a male, with known ischemic heart disease, collapsing after sudden back pain. A computed tomography scan demonstrated an aneurysm of the right sinus of Valsalva. The surgical repair aimed to exclude the aneurysm, preserving and reconstructing the aortic root.

## Introduction

Isolated aneurysm of just one of the three Valsalva's sinuses is a rare condition reported in literature with an incidence of 0.09 to 1.5%. 1

It is a consequence of a weakness of the elastic lamina at the junction of the aortic media and the annulus fibrosis and can be either congenital or acquired.

When congenital, this condition is generally associated with connective tissue disorders as Marfan's or Ehlers–Danlos syndrome 2 or with bicuspid aortic valve. 3

When acquired, the weakness of the elastic lamina may be related to infections, atherosclerosis, cystic medial necrosis. An aneurysm of the sinus of Valsalva might also be caused by chest trauma, vasculitis, or happens as complication of surgical or diagnostic procedures. 4 5

## Case Presentation

A 68-year-old man, with an established left anterior descending coronary artery disease, had initially declined surgical revascularization, citing his asymptomatic status.

The patient subsequently presented at a remote hospital as an emergency case, having collapsed at home after complaining of sudden onset of back pain. He was resuscitated and the initial assessment demonstrated electrocardiographic and biochemical evidence of posterior myocardial infarction.

Given the new symptom of back pain, the patient underwent a nongated computed tomography (CT) scan to exclude any acute aortic pathology. There was no evidence of dissection, but the CT scan demonstrated a voluminous aneurysm of the aortic root and the patient was transferred to a cardiothoracic center for consideration of urgent cardiac surgical intervention.

**Fig. 1:**
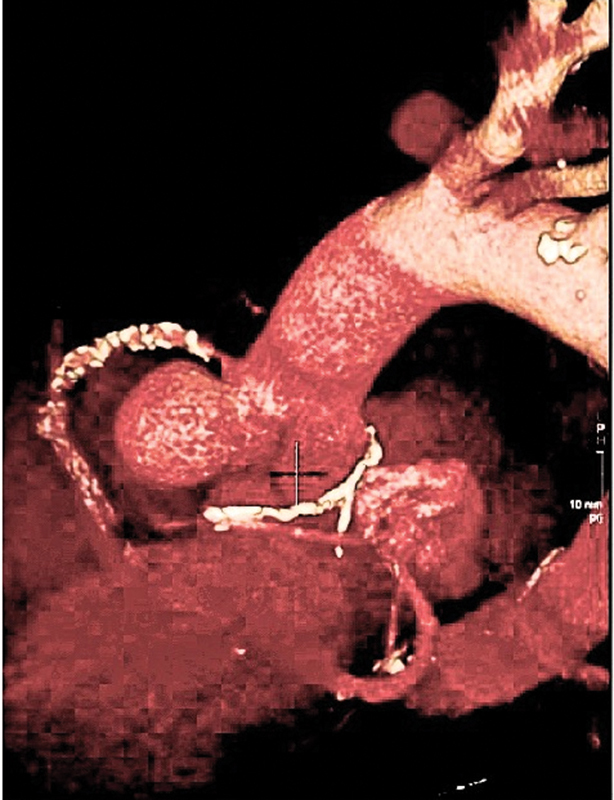
Saccular aneurysm arising from the right coronary cusp at the 12 o'clock position.

The patient underwent subsequently a gated CT aortogram and CT coronary angiography, and this clarified the size and nature of the aortic root aneurysm ( Fig. 1 ). The aneurysm involved the right sinus of Valsalva at the 12 o'clock position; it was separate from the origin of the right coronary artery which was stretched over the aneurysm. The aneurysm measured 31 mm × 33 mm × 29 mm and was fully opacified with contrast. There was no mural thickening of the aortic wall, no obvious flow-limiting disease of the right coronary artery and no acute aortic pathology.

Transthoracic echocardiography demonstrated significant turbulence in the right ventricular outflow tract but no other abnormality, and the cardiac function was preserved.

The patient was taken to the operating room, where the imaging findings were confirmed. The aneurysm arose just below the ostium of the right coronary artery, the course of which was stretched over the aneurysm with slit-like compromise of the lumen. There was also partial compression of the right ventricular outflow tract.

The repair began by transecting the ascending aorta just above the sinotubular junction. The right sinus was excised and a right coronary button was fashioned. A neosinus was tailored using a Dacron patch that was cut to the size of the excised sinus. The neosinus was sutured in place using 5–0 Prolene and this was then used as a running suture to reapproximate the ascending aorta. The right coronary button was reimplanted into the neosinus and the operation was completed with a bypass graft to the left anterior descending coronary artery using a pedicled left internal mammary.

**Fig. 2:**
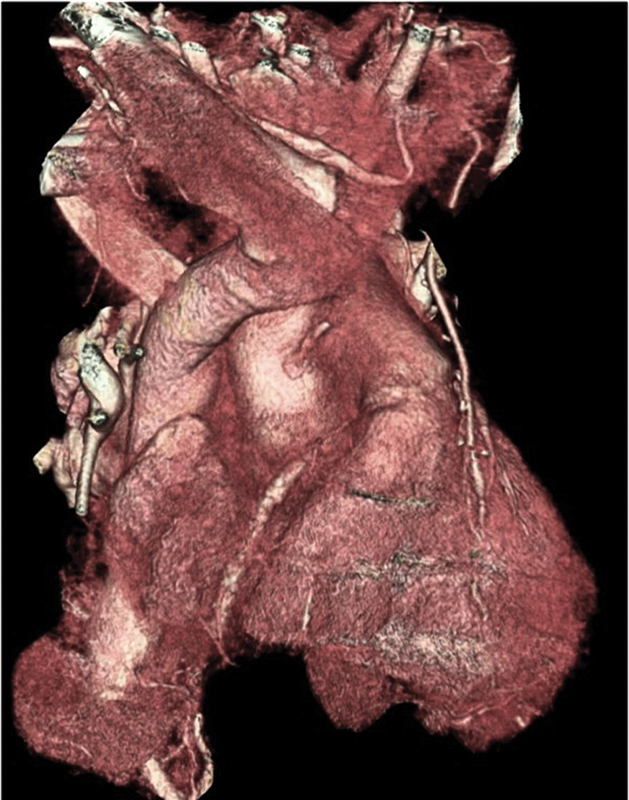
Postoperative computed tomography scan showing good result of the surgical repair.

The patient had an uncomplicated postoperative course. A predischarge gated CT aortogram demonstrated satisfactory repair with the aortic root, assuming normal appearance and dimensions ( Fig. 2 ).

## Discussion

Isolated sinus of Valsalva aneurysm as asymmetrical root pathology, is a rare cardiac abnormality with a reported incidence between 0.09 and 0.15%. 1 The condition can be either congenital or acquired. 2 3 4 5

The “unruptured” sinus of Valsalva aneurysm is usually silent and often a serendipitous finding due to its secondary effect on adjacent anatomical structures. In the present case, the right coronary artery was stretched, mimicking an acute coronary syndrome. On occasion, the first symptom is that of embolization due to thrombus formation within the aneurysm. Rupture of the aneurysm is catastrophic if external but can be equally problematic if the rupture is internal, creating a communication between the aorta and one of the cardiac chambers, most commonly the right atrium or right ventricle. 6

The present case illustrates an atypical presentation of a previously silent aneurysm of the right sinus of Valsalva being discovered only when the patient presented with the symptoms and signs that could be related to an acute coronary syndrome or an acute aortic pathology.

The patient had undergone coronary angiography 1 year prior to presentation but no aortogram or ventriculogram was performed at that time. Thus, repeat imaging was critical to make the diagnosis.

Incidentally, iatrogenic damage to the aortic wall from the previous coronary angiogram was considered, but intraoperatively the aortic wall appeared pristine and there was no evidence of a previous penetrating injury.

This would suggest that in the present case the “silent” isolated right sinus of Valsalva aneurysm was nontraumatic in nature and possibly growing in size; it became fortuitously apparent with the emergency clinical presentation.

The present case is a good example of a rare but noteworthy condition that is insidious in nature, often becoming apparent either when it reaches a significant size or as an incidental finding in patients with concomitant pathology.

Operative strategy should focus on exclusion of the aneurysm and reconstruction with the aim of preserving the aortic root where possible. Surgical repair remains the mainstay of treatment. Direct closure has been reported in small defects, and patch repair is preferred in larger defects to achieve tension-free repair. 7 Endovascular interventions have also been reported with success in defects smaller than 0.8 cm. 8

Repair or replacement of the aortic valve will be required if there is involvement of the valve leaflets with consequent valve dysfunction.

## References

[1] Bricker A O Avutu B Mohammed T L Valsalva sinus aneurysms: findings at CT and MR imaging Radiographics 2010 30 01 99 110 20083588 10.1148/rg.301095719

[2] Ott D A Aneurysm of the sinus of valsalva Semin Thorac Cardiovasc Surg Pediatr Card Surg Annu 2006 165 176 16638563 10.1053/j.pcsu.2006.02.014

[3] Moustafa S Mookadam F Cooper L Sinus of Valsalva aneurysms–47 years of a single center experience and systematic overview of published reports Am J Cardiol 2007 99 08 1159 1164 17437748 10.1016/j.amjcard.2006.11.047

[4] Seo K W Park J S Sinus of Valsalva aneurysm and multiple aortic aneurysms provoked by viral myocarditis Korean Circ J 2019 49 02 194 196 30693683 10.4070/kcj.2018.0309 PMC6351280

[5] Cohen R Mena D Carbajal-Mendoza R Arole O Mejia J O A case report on asymptomatic ascending aortic dissection Int J Angiol 2008 17 03 155 161 22477421 10.1055/s-0031-1278301 PMC2727764

[6] Le D D Orrego C M Maragiannis D Chang S M An unusual case of right-sided heart failure caused by giant sinus of Valsalva aneurysm obstructing right ventricular outflow tract Eur Heart J 2014 35 39 2721 24935920 10.1093/eurheartj/ehu234

[7] Yan F Huo Q Qiao J Murat V Ma S F Surgery for sinus of valsalva aneurysm: 27-year experience with 100 patients Asian Cardiovasc Thorac Ann 2008 16 05 361 365 18812342 10.1177/021849230801600504

[8] Kuriakose E M Bhatla P McElhinney D B Comparison of reported outcomes with percutaneous versus surgical closure of ruptured sinus of Valsalva aneurysm Am J Cardiol 2015 115 03 392 398 25488356 10.1016/j.amjcard.2014.11.013

